# Pilocarpin in Asthma

**Published:** 1881-01

**Authors:** 


Pilocarpin in Asthma.—Pilocarpin is the latest asthma remedy. One-fourth or one-third of a grain hypodermically injected, or the same dose by the mouth, daily or oftener, is the method recommended. It should be remembered that it is a powerful heart depressant and should be watched.—*Arkansas Medical Monthly.*

